# PCNP promotes ovarian cancer progression by accelerating β‐catenin nuclear accumulation and triggering EMT transition

**DOI:** 10.1111/jcmm.15491

**Published:** 2020-06-16

**Authors:** Pengzhen Dong, Hao Fu, Lin Chen, Shihui Zhang, Xin Zhang, Huimin Li, Dongdong Wu, Xinying Ji

**Affiliations:** ^1^ Henan International Joint Laboratory for Nuclear Protein Regulation School of Basic Medical Sciences Henan University Kaifeng China; ^2^ Huaihe Hospital Henan University Kaifeng China; ^3^ Department of Histology and Embryology Institute of Biomedical Informatics Cell Signal Transduction Laboratory Bioinformatics Center School of Basic Medical Sciences Henan University Kaifeng China; ^4^ Institute for Innovative Drug Design and Evaluation School of Pharmacy Henan University Kaifeng China; ^5^ Henan International Joint Laboratory for Nuclear Protein Regulation School of Stomatology Henan University Kaifeng China; ^6^ Kaifeng Key Laboratory of Infection and Biological Safety Henan University College of Medicine Henan University Kaifeng China

**Keywords:** epithelial‐mesenchymal transition, migration, ovarian cancer, PEST‐containing nuclear protein, Wnt, β‐catenin

## Abstract

Ever reports showed that PCNP is associated with human cancers including neuroblastoma and lung cancer. However, the role and underlying molecular mechanism of PCNP in ovarian cancer have not been plenty elucidated. Herein, we first investigated the expression of PCNP in ovarian cancer tissues and cells, the effects of PCNP in ovarian cancer proliferation, apoptosis, migration and invasion, and determined the molecular mechanism of PCNP in ovarian cancer progression. The results indicated that PCNP was significantly overexpressed in human ovarian cancer tissues and cells, and related to poor prognosis in ovarian cancer patients. In addition, we also detected that PCNP promoted ovarian cancer cells growth, migration and invasion, as well as inhibited ovarian cancer cells apoptosis. Mechanistically, PCNP binding to β‐catenin promoted β‐catenin nuclear translocation and further activated Wnt/β‐catenin signalling pathway. Moreover, PCNP regulated the expression of genes involved in EMT and further triggered EMT occurrence. Conclusionally, PCNP may promote ovarian cancer progression through activating Wnt/β‐catenin signalling pathway and EMT, acting as a novel and promising target for treating ovarian cancer.

## 1 INTRODUCTION

Ovarian cancer (OC) is the leading cause of death in female reproductive system malignancies and has a 5‐year survival rate of ~47%.^1^, ^2^ There are approximately 239 000 new cases and 152 000 deaths worldwide annually with regard to this disease.^3^, ^4^, ^5^ Only 15% of ovarian cancer are diagnosed at clinically early stage, and most patients carry metastatic tumours when they are diagnosed as ovarian cancer patients, further acquire poor prognosis and high mortality rate. So far, the treatment for most ovarian cancers may be surgery and cytoreduction,^6^ repeated treatment and drug‐resistance also lead to a low 5‐year survival. Thus, it is imperative to explore novel and effective biomarker, and underlying molecular mechanism of ovarian cancer metastasis, further supplying new therapeutic interventions for ovarian cancer.^7^

PEST‐containing nuclear protein (PCNP) is firstly identified in the nucleus through database mining.^8^ Zhan et al and Sun et al, respectively, depicted the higher RNA level of PCNP in Myeloma and Central Nervous system (CNS) cancer compared with non‐tumour normal, without any biological function exploration for PCNP in cancers.^9^, ^10^ Recently, Wang DY et al summarized that PCNP promotes cell proliferation, migration and invasion in lung adenocarcinoma; however, Wu DD et al considered that PCNP inhibited human neuroblastoma progression.^11^, ^12^ However, the role of PCNP in OC tumorigenesis and progression remains to be elucidated. Herein, we investigated the effects and the underlying mechanism of PCNP in ovarian cancer.

Wnt/β‐catenin signalling pathway, which is critically involved in both development and homeostasis of tissues, is indispensable in regulating a variety of cellular biological activities.^13^ Abnormal activation of this signal pathway has been implicated in various tumours including ovarian cancer, which can drive a variety of tumorigenic functions including regulation of transformation, cell proliferation and invasion.^13^, ^14^, ^15^ In the absence of WNT ligand stimulation, β‐catenin is constitutively phosphorylated via interaction with glycogen synthase kinase 3β (GSK3β) in a destruction complex composed of scaffold proteins Axin and adenomatous polyposis coli (APC), leading to ubiquitin‐mediated degradation of β‐catenin and inactivation of the signalling.^16^ Recently, studies exhibit that mRNA of PCNP has been detected in some cancer cells, including U‐937 myeloid leukaemia cells, HepG2 hepatoma cells and HT‐1080 fibrosarcoma cells,^17^ suggesting the potential and promising relation of PCNP to other cancer cells including ovarian cancer cells. The tumour‐related characteristic of PCNP and the vital role of Wnt/β‐catenin in ovarian cancer prompt us to be interested in potential functional connections between PCNP and β‐catenin.

In ovarian cancer, the dysregulation of Wnt/β‐catenin can induce the epithelial‐mesenchymal transition (EMT).^18^ EMT, plays a critical role in embryonic development and also involved in cancer progression and metastasis,^19^, ^20^ contributes to new tumour cell properties required for invasiveness and vascular intravasation during metastasis.^21^, ^22^ It is a changeable process during which the epithelial cells lose epithelial properties and acquire mesenchymal characteristics by disassembly of cell‐cell junctions, loss of cell polarity, and reorganization of the cytoskeleton, thereby promoting cells to acquire increased motility.^23^ EMT can be defined according to EMT‐associated markers, such as mesenchymal‐specific markers, epithelial‐specific markers and transcription factors.^19^

In this study, we examined the effect of PCNP on OC cells proliferation, migration, invasion and apoptosis, showing that Wnt/β‐catenin pathway was severely affected by PCNP overexpression, and further regulate ovarian cancer tumorigenicity and development for ovarian cancer. Summatively, PCNP may have the properties to be used as a new target in the prediction and treatment of ovarian cancer.

## 2 METHODS

### 2.1 Cell lines and culture condition

Human ovarian cancer cell lines SK‐OV‐3 and A2780 were purchased from Shanghai Biological Technology Co., Ltd. enzyme research (Shanghai, China). Human normal ovarian epithelial cell IOSE80 was purchased from Otwo Biotech (Shenzhen, China). A2780 were maintained in RPMI 1640 medium (Corning, China) containing 10% foetal bovine serum (FBS, Biological Industries, Israel); SK‐OV‐3 were maintained in McCOY’s 5A medium (JiNuo Biology, China) and IOSE80 were maintained in DMEM medium (Corning, China) containing 10% fetal bovine serum (FBS, Biological Industries, Israel). Cells were grown in incubator with a humidified atmosphere of 5% CO2 at 37°C.

### 2.2 Construction of cell models

PCNP expression plasmid and empty vector, the PCNP shRNA (sh‐PCNP group) and scramble shRNA (sh‐Scb group) were purchased from Genechem (Shanghai, China) and were transfected into OC cells with Lipofectamine 3000 Transfection Reagent (Invitrogen, Shanghai, China) to construct stable cell lines. They were screened, respectively, by G418 (Solarbio, Shanghai, China) at a concentration of 800 μg/mL and puromycin (Solarbio, Shanghai, China) at a concentration of 2 μg/mL. The un‐transfected tumour cells were used as controls. Seventy‐two hours post‐transfection, the localization of PCNP within tumour cells was detected under a fluorescent microscope (Eclipse Ti, Nikon, Melville, NY, USA).

### 2.3 Real‐time fluorescence quantitative PCR (qPCR)

The relative expression level of PCNP was measured by RT‐PCR. The total RNA was extracted using the Trizol reagent (Takara, Dalian, China) and then reverse‐transcribed into cDNA by using a Revert Transcription kit (Abm, Canada). The forward and reverse primers used for PCNP DNA amplification were 5′‐ATAGGATCCAAAATGGCGGACGGGAAGGCG‐3′ and 5′‐CCGAA GCTTTTAATTGTCTT GGTCATGGAC‐3′. Housekeeping gene GAPDH was used as an internal reference and forward and reverse primers were as follows: 5′‐TATGACAACGAATTTGGCTACAG‐3′ and 5′‐GATGGTA CATGACAAGGTGC‐3′. The reaction system of RT‐PCR is 10μL including TB Green for 5 μL (Takara, Dalian, China), passive reference Dye for 0.21 μL. 0.5 μL of forward and 0.5 μL of reverse primers (10 μM), cDNA for 2 μL (200 ng/μL) and ddH_2_O for 2 μL. The reaction procedure is as follows: denaturation at 95°C for 5 min; followed by 35 cycles of 95°C for 30 second, annealing at 60°C for 30 second, and a final extension at 72°C for 30 second, and then at 72°C for 10 min. Data were analysed using the 2^−ΔΔCt^ method, and the relative mRNA expression levels were normalized against GAPDH.

### 2.4 Cell proliferation assay

The 5‐ethynyl‐2′‐deoxyuridine (EdU) incorporation assay was performed using the Cell‐Light EdU Apollo 567 In Vitro Imaging Kit (RiboBio, Guangzhou, Guangdong, China). A2780 and SK‐OV‐3cells were introduced into a 12‐well plate at 4 × 10^5^ cells/well and incubated with 10 µM EdU for 2 hour followed by culture for 24h. Then, cells were fixed in 4% paraformaldehyde at room temperature for 30 minutes and then treated with 0.5% Triton X‐100 solution at room temperature for 15 minutes. Next, 200μL Apollo solution was added into every well and incubated at room temperature for 30 minutes under dark. Cells were observed under a fluorescent microscope (Eclipse Ti, Nikon, Melville, NY, USA). Cell proliferation rate (%) = (EdU‐positive cells)/(total number of cells) × 100.

### 2.5 Colony formation assay

Cells were introduced into a 6‐well plate at 5 × 10^2^ cells/well and incubated for a week, after washing by phosphate‐buffered saline (PBS) for 3 times, the colonies were subjected into methanol at room temperature for 15 minutes. Then, 0.1% crystal violet was added into each well and incubated for 30 minutes at room temperature. After that, wash the plate by water gently and air‐dried at room temperature. The plate was photographed under an Olympus CKX41 microscope and then measured using Image J software.

### 2.6 Wound healing assay

Cells were seeded in wells for 12 hour at 37°C under 5% CO_2_. A sterile micropipette tip was used to scratch the cells and then washed with PBS. The migration distance was photographed by Olympus CKX41 microscope on 0, 12, 24 hour and then measured with Image J software (National Institute for Health, Bethesda, MD, USA). Migration rate (MR) was calculated by the formula of MR (%) = [(A − B)/A] × 100(A is the width of 0 hour and B is the width of 24 hour).

### 2.7 Soft agar assay

Cells were suspended in 0.6% agarose and medium supplemented with 20% FBS at 1 × 10^4^ cells/well, and the mixture was seeded in 6‐well plates containing a basal layer of 1.2% agarose. The top layer is added with medium for twice per week to prevent drying. After 2 weeks of routine culture, colonies were photographed under an Olympus CKX41 microscope. Only colonies larger than 0.1 mm in diameter were counted.

### 2.8 Migration and invasion assays

Cell migration and invasion were evaluated using transwell chambers with 8μm pores. 5 × 10^4^ cells/well in serum‐free medium were seeded into the upper chamber uncoated or coated with Matrigel (BD Biosciences, San Jose, CA, USA). The medium containing 20% FBS was added to the lower chamber at 600 μL/well. After incubation for 24 hour, remaining cells were scrubbed off with cotton swabs, while cells on the bottom surface of the membrane were fixed with methanol and stained with 0.1% crystal violet. The cell number was counted using a Zeiss Axioskop 2 plus microscope (Carl Zeiss, Thornwood, NY, USA).

### 2.9 Apoptosis assay

Terminal‐deoxynucleotidyl transferase‐mediated nick end labelling (TUNEL) assay was conducted using an In Situ Cell Death Detection Kit (Beyotime Biotechnology, Shanghai, China) according to the manufacturer's protocols. A2780 and SK‐OV‐3 cells were introduced into a 12‐well plate at 2 × 10^5^ cells/well and incubated for 24 hour. After washing by PBS, cells were fixed in 4% paraformaldehyde at room temperature for 30 minutes and then treated with 1% Triton X‐100 solution for 10min at room temperature. Then, 200μL TUNEL solution was added into each well and incubated at 37℃ for 80 minutes under dark and stained with fluorescent dyes. 4′, 6‐diamidino‐2‐phenylindole (DAPI) was used to stain the cell nuclei at room temperature for 5min under dark. Cells were observed under a fluorescent microscope (Eclipse Ti, Nikon, Melville, NY, USA). The percentage of TUNEL‐positive cells was calculated using Image J software.

### 2.10 Immunoblottingting analysis

Cells were collected and added appropriate amount of IP lysate (Beyotime, Shanghai, China) for total protein; and nuclear and cytosolic isolation kit (KeyGEN BioTECH, China) for nuclear and cytosolic protein. Equal mass of protein in each sample was separated on SDS‐PAGE and transferred to PVDF membrane (Millipore, Merck KgaA, Darmstadt, Germany). Then, the membrane was blocked in 5% skim milk at room temperature for 1 hour, after that the membrane was incubated with primary antibody (GAPDH, LaminB1, β‐Tubulin, cleaved‐Caspase8, 9, PARP, Bcl‐2, Bcl‐xl, Bad, PCNP, ZEB1, N‐Cadherin, E‐Cadherin were bought from Proteintech, Wuhan, China; AKT, p‐AKT, PI3K, p‐PI3K, GSK3β, p‐GSK3β, β‐catenin were bought from Cell Signaling Technology, MA, USA) at 1:1000 overnight at 4℃. Next, the membrane was incubated with HRP‐conjugated Goat anti‐rabbit IgG (H + L) secondary antibody (1:5000, Proteintech, Wuhan, China) at room temperature for 60min followed by detection of protein band after addition of ECL chemiluminescence (Meilunbio, Dalian, China).

### 2.11 Tumour xenograft formation assay

All animal procedures were manipulated with the approval of the Medical and Research Ethics Committee of Henan University Medical School. Xenograft tumour model was performed in female BALB/c‐nude mice (3‐4 weeks of age), which were purchased from Beijing Vital River Laboratory Animal Technology Co., Ltd. China. A total of 2 × 10^7^ cells stably transfected of SK‐OV‐3 and A2780 were subcutaneously injected into the right armpit of the mice (n = 5 per group). After a week, mouse weight and tumour volumes were measured every 2 day with a digital caliper by the following formula: Volume = (length × width^2^)/2. Mice were killed 3 weeks after the initiation of treatment, and the tumour weight and volume were evaluated. Tumour volume double time (TVDT) = (*T* − *T*_0_)*log2/log(*V*_2_ − *V*_1_) (*T* is the last day to measure the volume, *V*_1_ is the first volume you measure, *V*_2_ is the last volume you measure); tumour inhibitory rate (%) = (W test − W control)/ W test*100% (W test is the weight of test groups and W control in the weight of control group).

### 2.12 Immunohistochemistry and HE assay

Four μm thick tissue sections were cut, dried, dewaxed and hydrated according to standard procedures. An EDTA (Solarbio, BeiJing, China) heat‐induced antigen retrieval process is then performed. Staining was optimized using markers (PCNP, CD31, Ki67, N‐cadherin and E‐cadherin) with Universal two‐step assay kit (mouse/rabbit reinforced polymer assay system) and DAB (Zhong Shan Jin Qiao, BeiJing, China).

### 2.13 Cycloheximide (CHX) assay

Cells were introduced into a 6‐well plate at 5 × 10^4^ cells/well and incubated for 12 hour, 400 μg cycloheximide (CHX, MedChemExpress, China) was added into 2 mL medium. CHX is a eukaryote protein synthesis inhibitor, produced by the bacterium Streptomyces griseus. CHX interferes with the translocation step in protein synthesis and is widely used in biomedical research to inhibit protein synthesis in vitro. Samples were collected at 0, 1, 2, 3, 4 hour and stored for Immunoblottingting to detect β‐catenin degradation.

### 2.14 Co‐immunoprecipitation (Co‐IP) assay

A pierce Co‐Immunoprecipitation (Co‐IP) Kit was purchased from Thermo Scientific (Shanghai, China). Then, all the procedures were followed by this kit. The bound proteins were analysed with Western blottingting using relevant antibodies.

### 2.15 Statistical analysis

All values were expressed as the mean ± standard deviation (SD). Two‐tailed Student's *t* test and ANOVA analysis were performed for statistical comparison. Asterisk indicates that the values are significantly different (*, #, *P* < .05; **, ##, *P* < .01).

## 3 RESULTS

### 3.1 PCNP was overexpressed and related to overall survival in ovarian cancer

**Figure 1:**
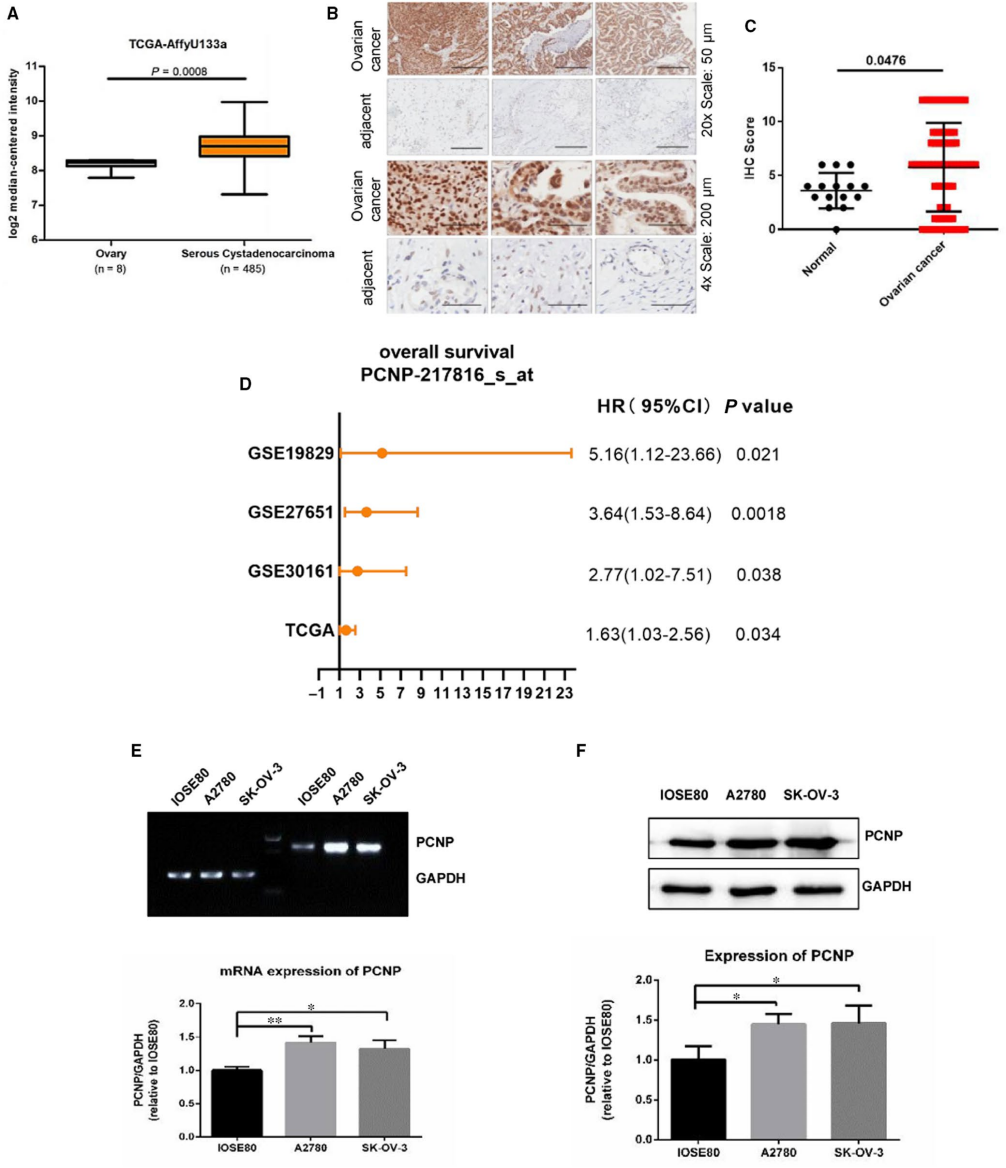
Expression and prognostic analysis of PCNP in OC tissues. A, The analysis of PCNP transcription level between normal and ovairan cancer according to TCGA data. B, C, Immunohistochemical analysis of OC microarray chip. D, Overall survival analysis of PCNP in ovarian cancer according to prognostic data of four cohorts including GSE19829, GSE27651, GSE30161 from GEO Datasets and TCGA data. E, F, mRNA and protein expression identification of PCNP in ovarian epithelial cell IOSE80 and OC cells SK‐OV‐3 and A2780 with PCR and Western blotting, GAPDH was used as the control

**Figure 2:**
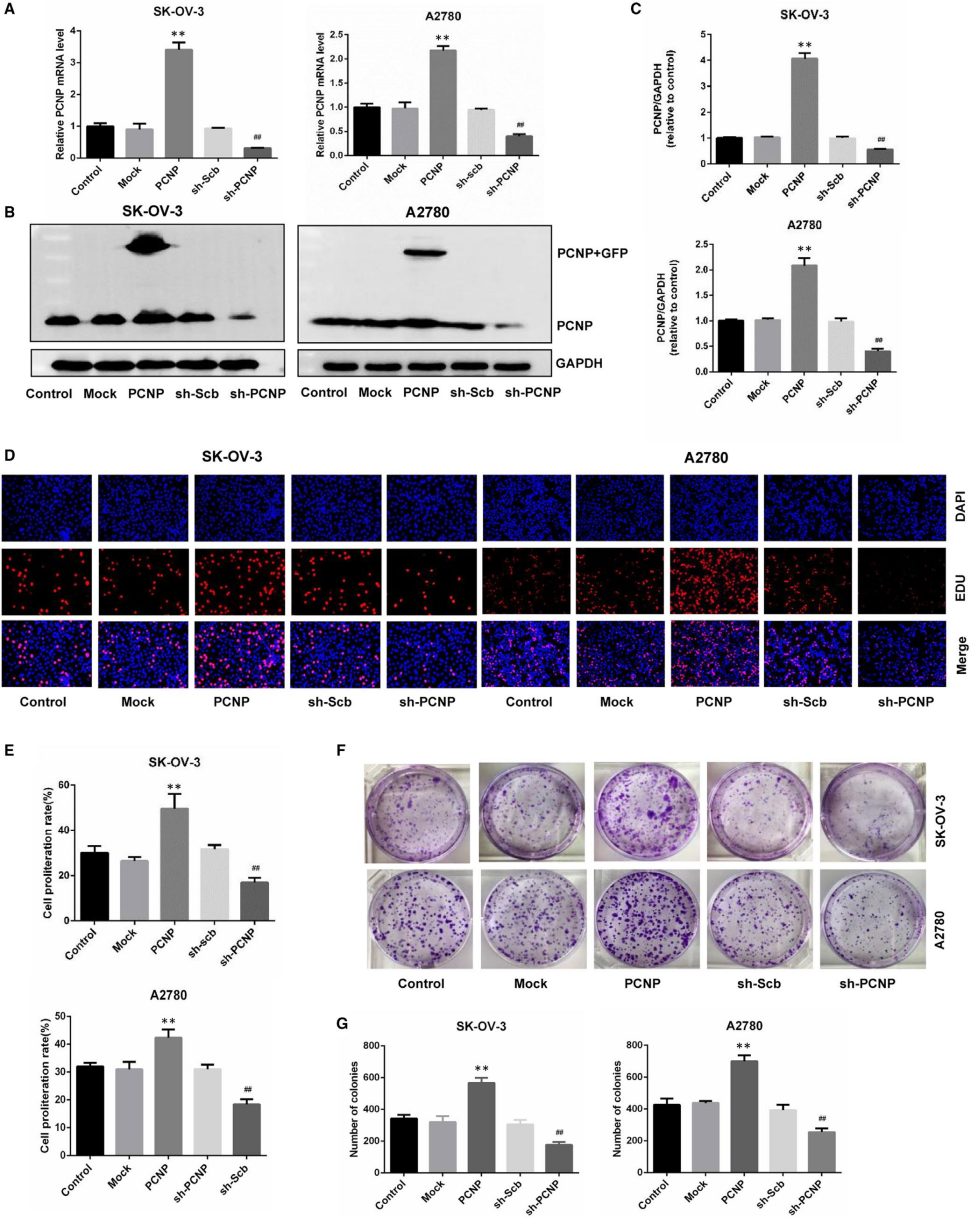
The effects of PCNP on the proliferation of OC cells. A, The mRNA level of PCNP in SK‐OV‐3 and A2780 was analysed by RT‐PCR. B, C, The protein analysis of over‐ and down‐regulation of PCNP in SK‐OV‐3 and A2780 cells by Western blotting normalized to the corresponding GAPDH level. D, DNA replication of SK‐OV‐3 and A2780 cells in normal and over‐ or down‐regulated PCNP group was analysed by EdU assay; original magnification 200×. E, The proliferation rate of each group was analysed. F, Clonogenic capacity was detected in SK‐OV‐3 and A2780 cells; original magnification 200×. G, The number of colonies was calculated. Data are presented as mean ± SEM of three independent experiments; ^*^ *P* < .05, ^**^ *P* < .01 compared with the Mock group; ^#^ *P* < .05, ^##^ *P* < .01 compared with the sh‐Scb group

To preliminary detect the association of PCNP with OC, its transcriptome data in the Cancer Genome Atlas (TCGA) was collected and identified, discovering that PCNP was significantly higher in OC than normal tissues (*P* < .001; Figure 1A). Moreover, immunohistochemical analysis of microarray chip including normal and OC patient samples showed the protein expression of PCNP in OC tissues was significantly increased, which is consistent to the identification using TCGA data (Figure 1B‐C). During the analysis of clinical and transcriptional data of OC in four GEO datasets, it was illustrated that patients with high PCNP level had a worse overall survival (OS) (Figure 1D). To clarify the overexpression of PCNP in cell levels, we also compared the mRNA and protein levels of PCNP in ovarian epithelial cell IOSE80 and two common OC epithelial cells SK‐OV‐3 and A2780. The results showed that the mRNA and protein level of PCNP in ovarian cancer cells were both significantly higher than that in IOSE80 cells (Figure 1E‐F). Then, we successfully constructed stabilized PCNP‐mediated OC cells (Figure 2A‐C). Western blotting results suggested the cell models could be used to perform further research around PCNP.

### 3.2 PCNP promoted ovarian cancer cells proliferation and suppressed its apoptosis

To evaluate the role of PCNP in OC cells proliferation, we conducted EDU assay and detected that the number of cells in the proliferative phase for overexpressed PCNP group was significantly higher than that of mock group, meanwhile, the PCNP knock‐down group was significantly lower than that of sh‐scb group (Figure 2D‐E), showing the proliferative capacity of PCNP in OC cells.

**Figure 3:**
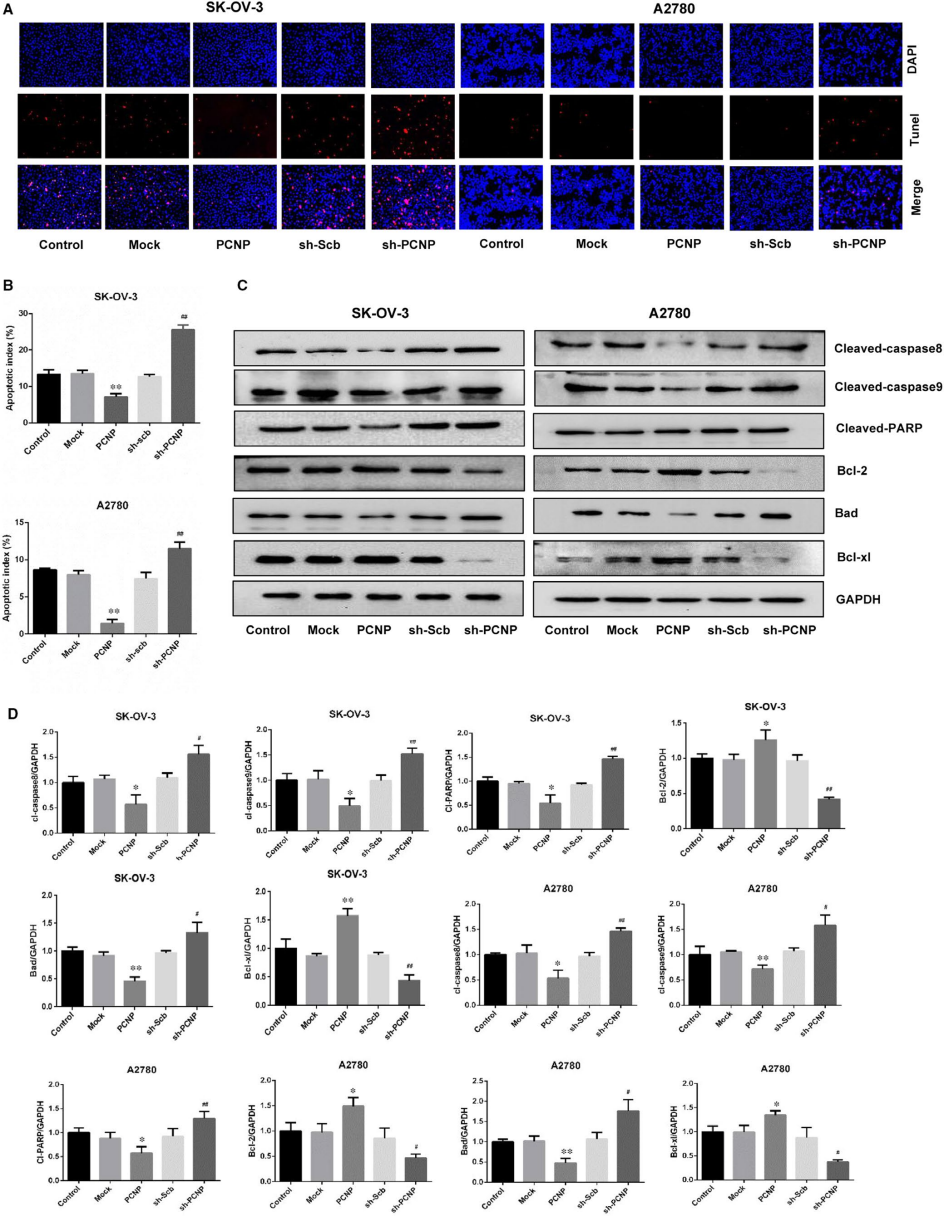
The effects of PCNP on apoptosis of OC cells. A, Apoptosis level of SK‐OV‐3 and A2780 cells was detected by Tunel staining; original magnification 200×. B, The calculation and analysis of apoptosis rate of SK‐OV‐3 and A2780. C. Western blotting analysis for the expression of cleaved‐caspas8, cleaved‐caspase9, cleaved‐PARP, bad, bcl‐2 and bcl‐xl in PCNP over‐ or down‐expressed group for SK‐OV‐3 and A2780 cells. D, The analysis of proteins mentioned in (C) was performed, normalized to the corresponding GAPDH level. Data are presented as mean ± SEM of three independent experiments; ^*^ *P* < .05, ^**^ *P* < .01 compared with the Mock group; ^#^ *P* < .05, ^##^ *P* < .01 compared with the sh‐Scb group

To further and visually clarify the function of PCNP to promote growth, we also conducted colony formation, and verified that PCNP promoted OC cells proliferation and PCNP knock‐down played the opposite role (Figure 2F‐G). Multi‐cellular organisms maintain tissue homeostasis by balancing proliferation and removal of cells including cancer cells via apoptosis. Here, TUNEL assays were used to investigate whether PCNP promotes OC cells proliferation was accompanied by the suppression of cells apoptosis. As a result, in PCNP overexpressed group, the percentage of apoptotic cells was significantly lower than that in mock group, while knock‐down PCNP group was higher than sh‐scb group (Figure 3A‐B). Results of Western blotting presented that PCNP up‐regulated Bcl‐2 and Bcl‐xl, which inhibited cell apoptosis, meanwhile, down‐regulated cleaved‐caspase8, 9, PARP, Bad, which promoted cell apoptosis, yet PCNP knock‐down had an opposite effect (Figure 3C‐D). Together, PCNP may possess the proliferative and anti‐apoptotic capacity in ovarian cancer.

### 3.3 PCNP promoted ovarian cancer migration, invasion and EMT

**Figure 4:**
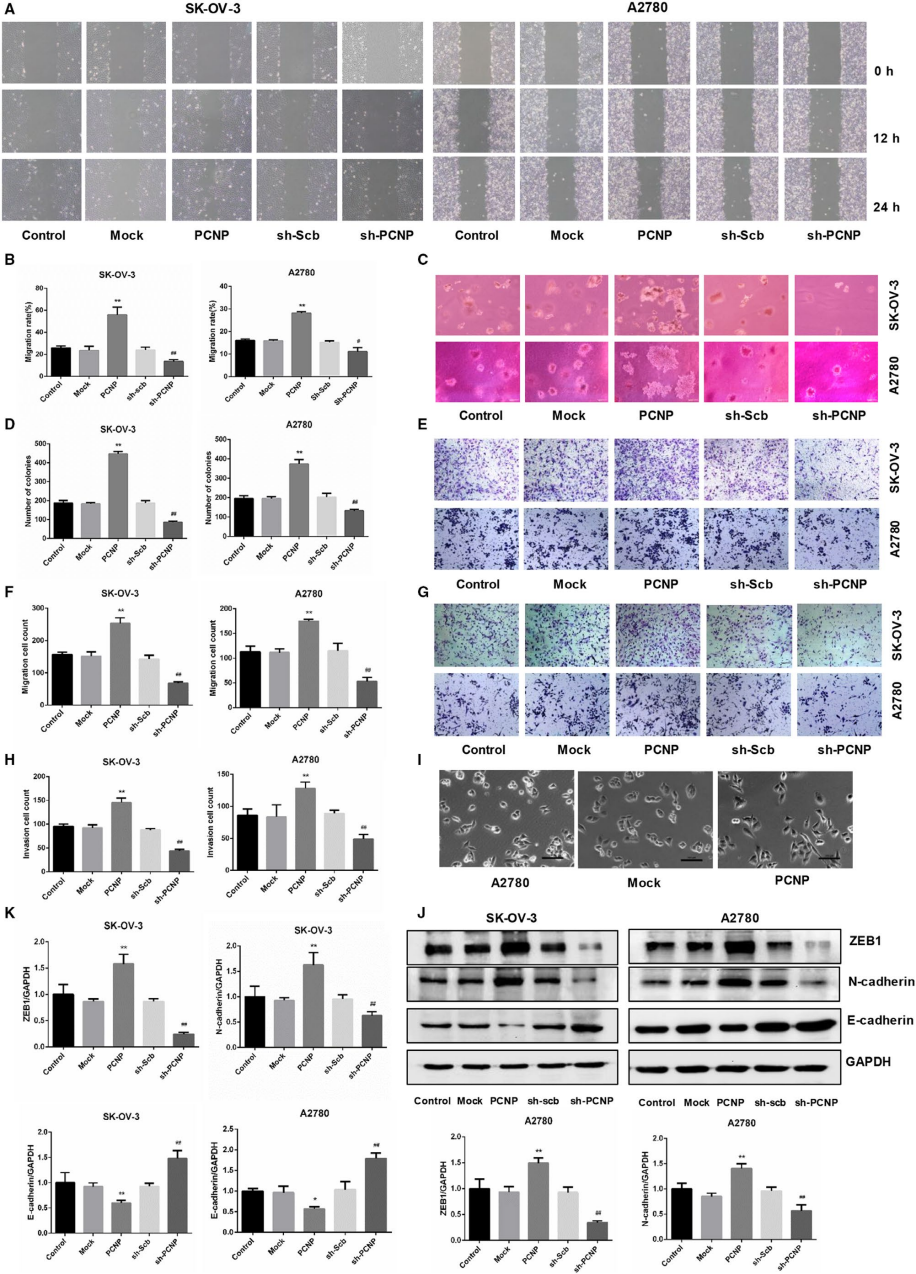
The effects of PCNP on the migration and invasion ability of OC cells. A, The effect of PCNP on the ability of cell migration was tested by wound healing assay; original magnification 100×. B, Migration rate of SK‐OV‐3 and A2780 was calculated. C, Soft agar test was performed to detect the anchorage‐independent survival of cells; original magnification 200×. D, The number of colonies was calculated. E, The migration ability of SK‐OV‐3 and A2780 cells was measured by transwell assay; original magnification 200×. F, The number of migrated cells was calculated. G, The invasion ability of SK‐OV‐3 and A2780 cells was measured by invasion assay; original magnification 200×. H, The number of invasive cells was calculated. I, Cell morphology of A2780 in each group. J, K, Western blotting analysis and densitometry analysis for the expression of epithelial‐mesenchymal transition (EMT) markers (ZEB1, N‐cadherin, E‐cadherin). GAPDH was used as the control. Data are presented as mean ± SEM of three independent experiments; ^*^ *P* < .05, ^**^ *P* < .01 compared with the Mock group; ^#^ *P* < .05, ^##^ *P* < .01 compared with the sh‐Scb group

Furthermore, to reveal the biological function of PCNP in OC, we investigate the effect of PCNP on OC cells migration and invasion. Results showed that the migration rate in PCNP overexpressed group for both SK‐OV‐3 and A2780 cells was significantly increased compared with corresponding mock group. However, PCNP knock‐down obviously lowered the migration rate (Figure 4A‐B). Consistently, soft agar colony formation assays and transwell assays were identified to present the same trend, together, demonstrating that invasion and migration of OC cells in vitro could be promoted by PCNP overexpression and inhibited by PCNP knock‐down (Figure 4C‐H). To visually investigate the morphological changes for OC cells with the conversion of the metastatic ability, observation was conducted using microscope and morphological changes named slender shape was detected, which was beneficial to cell metastasis and invasion and was typically characteristic of EMT (Figure 4I). To further identify the association of PCNP with EMT in OC cells, the protein expression of EMT‐related markers such as N‐cadherin, E‐cadherin and ZEB1 were checked, results in the Figure 4J‐K showed that PCNP significantly increased protein expression of mesenchymal markers ZEB1, N‐cadherin and decreased it of epithelial marker E‐cadherin, which were just also features of EMT. Summatively, PCNP could promote OC cells migration and invasion and EMT in vitro.

### 3.4 PCNP interacted to β‐catenin and increased nuclear accumulation of β‐catenin

**Figure 5:**
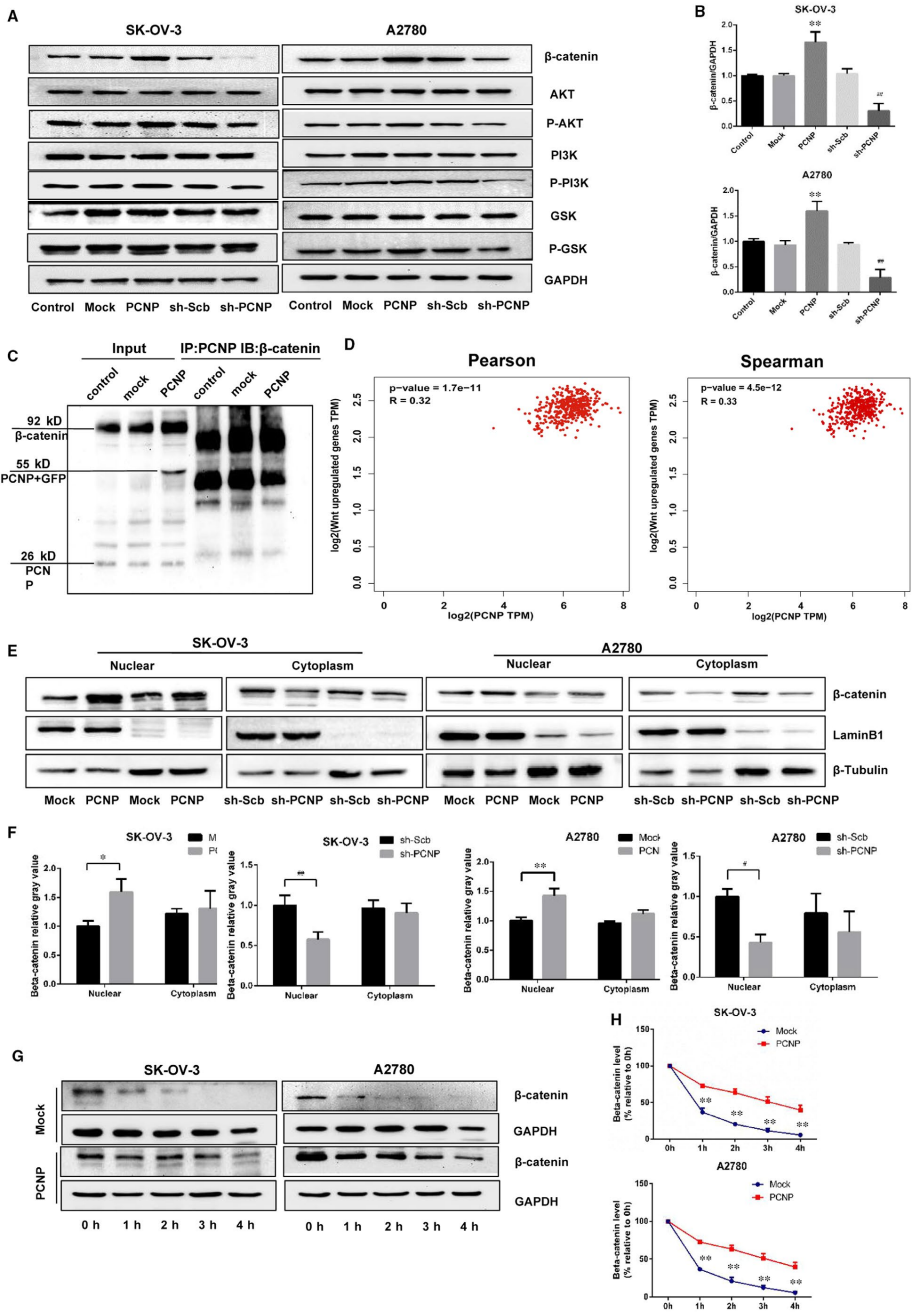
The identification of relation of PCNP with β‐catenin. A, Western blottingting analysis for the expression of AKT, P‐AKT, PI3K, P‐PI3K, GSK‐3β, P‐GSK‐3β, β‐catenin. B, The densitometry analysis of proteins shown in (A) was performed, normalized to the corresponding GAPDH level. C, Co‐IP assays reveal the association between PCNP and β‐catenin in A2780 cells. Inputs were used as control. CO‐IP was performed using antibodies as indicated. D, The correlation analysis of PCNP with Wnt/β‐catenin pathway up‐regulated genes according to GEPIA database. E, Expression of β‐catenin in the nucleus and cytoplasm of each group of SK‐OV‐3 and A2780. F, The densitometry analysis of β‐catenin was performed, normalized to the corresponding LaminB1 (nucleus) and β‐Tubulin (cytoplasm) level. G, β‐catenin degradation was investigated by cycloheximide (CHX). H. The densitometry analysis of β‐catenin in 0, 1, 2, 3, 4 h was performed, normalized to the corresponding GAPDH. Data are presented as mean ± SEM of three independent experiments; ^*^ *P* < .05, ^**^ *P* < .01 compared with the Mock group; ^#^ *P* < .05, ^##^ *P* < .01 compared with the sh‐Scb group

Wnt/β‐catenin and PI3K/Akt signalling pathways appear to play an important role in ovarian cancer. To expound the precisely potential mechanism of PCNP in OC progression, Western blotting was performed and resulted in that PCNP facilitated the up‐regulation of β‐catenin (Figure 5A‐B). However, total proteins of PI3K, Akt and GSK3β were not affected by PCNP, moreover, PCNP also scarcely regulated phosphorylated Akt, phosphorylated PI3K and phosphorylated GSK3β. To further confirm the association of β‐catenin with PCNP, we conducted Co‐IP assay and indicated PCNP could combine to β‐catenin (Figure 5C). Consistently and interestingly, the positive correlation of PCNP and Wnt/β‐catenin up‐regulated genes listed in File1 according to NetPath‐Signal Transduction Pathways database was also obtained from GEPIA database (Figure 5D). In Wnt/β‐catenin signal, the accumulation of β‐catenin promotes its nuclear translocation and further activates the downstream genes. As shown in Figure 5E‐F, results demonstrated that PCNP overexpression increased the accumulation of β‐catenin in the nucleus in both SK‐OV‐3 and A2780 cells. To explore the potential mechanism of PCNP‐mediated regulation of β‐catenin expression, we performed protein stability assays. As CHX is widely used to inhibit protein synthesis in eukaryotic cells, so we use it to detect the stability of β‐catenin. The half‐life of β‐catenin was significantly increased in the PCNP overexpression group compared with the mock group within 0‐4h after CHX treatment (Figure 5G‐H), synthetically, suggesting that PCNP could interact to β‐catenin and obstruct its degradation, further increase β‐catenin nuclear accumulation.

### 3.5 PCNP promoted ovarian cancer cell proliferation and metastasis in vivo

**Figure 6:**
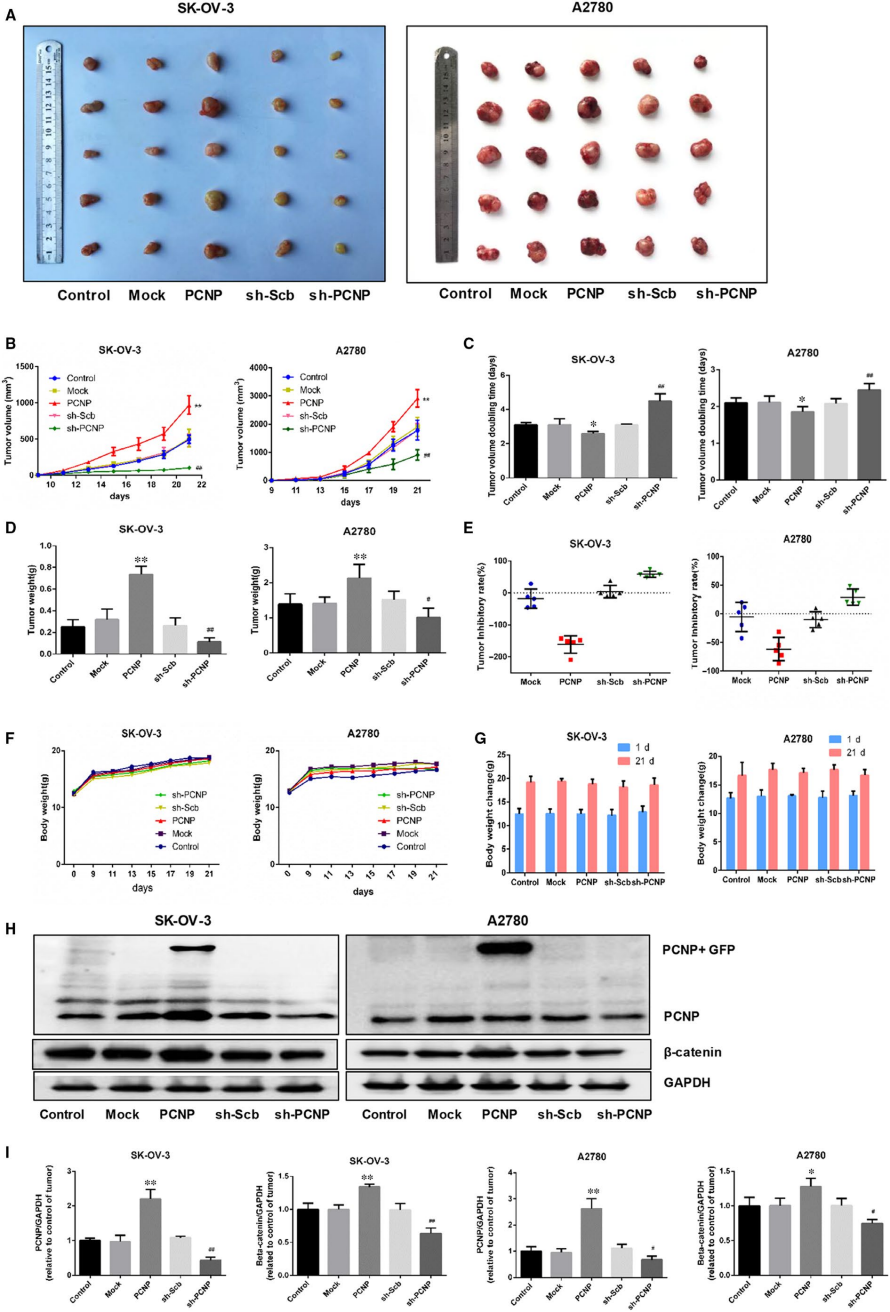
Effects of PCNP on the growth of SK‐OV‐3 and A2780 cells in vivo. A, Representative xenograft tumours taken from different groups of nude mice. B, C, The tumour volume of each group was measured every day, and the TVDT was calculated by the graph shown above. D, E, The tumours were weighed and the inhibition rates of tumour growth were calculated by the formula shown above. F, G, The bodyweight change curve of each group during the experiment and the bodyweight of each group on the first day (day 0) and the last day (day 21). H, Western blottingting analysis for the expression of PCNP and β‐catenin in tumours in nude mice of SK‐OV‐3 and A2780. I, The densitomsis of PCNP and β‐catenin was performed, normalized to the corresponding GAPDH level. Data are presented as mean ± SEM of three independent experiments; ^*^ *P* < .05, ^**^ *P* < .01 compared with the Mock group; ^#^ *P* < .05, ^##^ *P* < .01 compared with the sh‐Scb group

**Figure 7:**
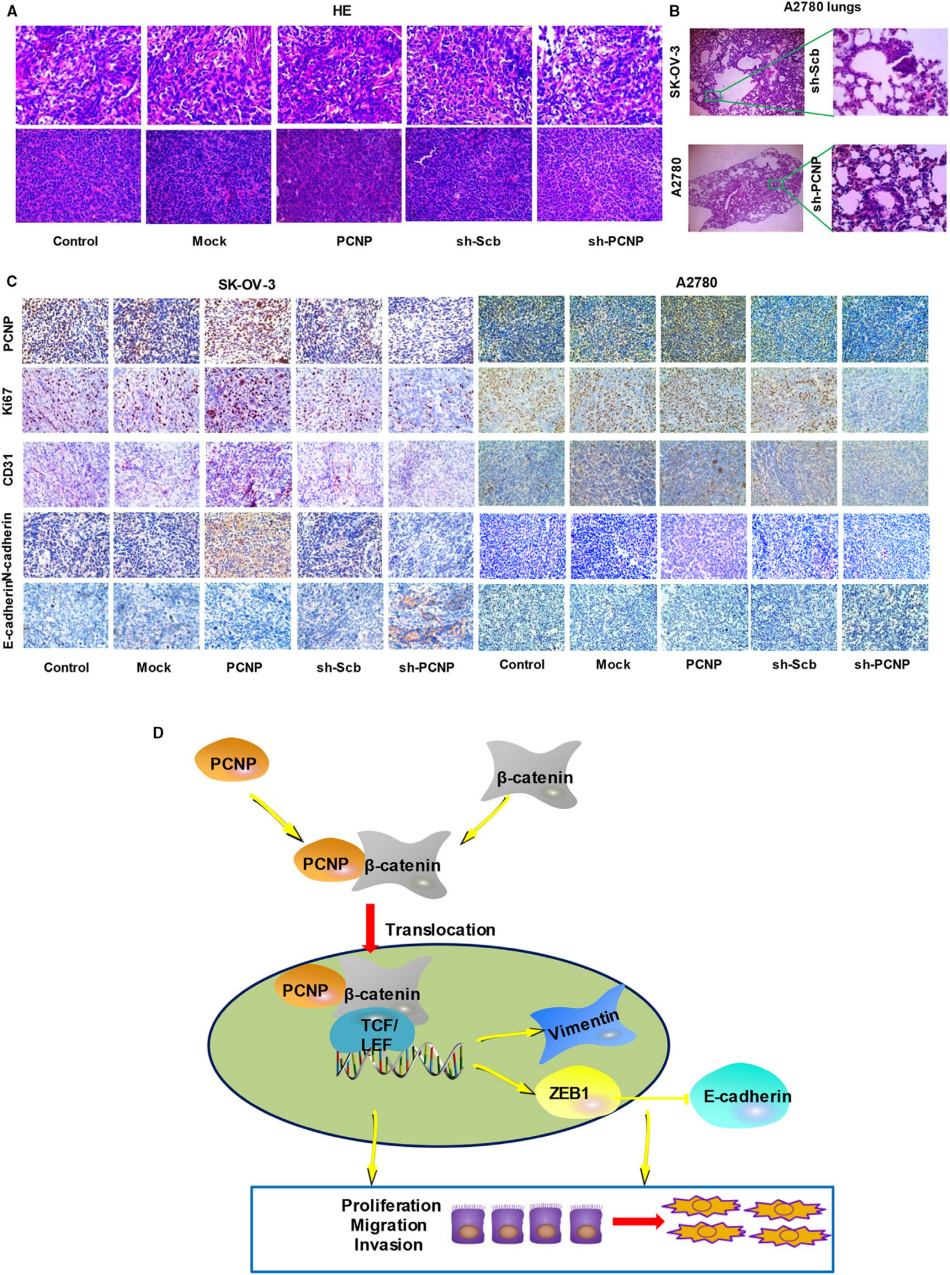
HE and Immunohistochemistry analysis of nude transplanted tumour and the identification of underlying mechanism of PCNP in ovarian cancer. A, HE staining of paraffin sections of nude mice in SK‐OV‐3 and A2780. B, Lung HE staining of sh‐scb and sh‐PCNP group (Lung tissue from nude mice bearing A2780 cells xenografts). C, Immunohistochemistry examination of PCNP, Ki67, CD31 and EMT‐related markers (N‐cadherin and E‐cadherin) expression in ovarian cancer tissues of nude transplanted tumour. D, PCNP accelerating the expression of nuclear β‐catenin and binding to β‐catenin activates Wnt signalling pathway and promotes ovarian cancer progression

To determine whether PCNP plays a role in ovarian cancer cells proliferation in vivo, we established a subcutaneous xenograft model of SK‐OV‐3 and A2780 cells in Balb/c nude mice. In the xenograft model, mice were monitored and weighed weekly and then killed in the third week. The PCNP overexpression group showed a rapidly increase in tumour volume and weight than that of mock group; PCNP knock‐down group had opposite effect (Figure 6A‐D). However, there was no difference in weight gain between nude mice in each group (Figure 6F‐G). The inhibition rate analyses showed the promotion of PCNP in tumour growth (Figure 6E). In heterogeneous tumours inoculated with both SK‐OV‐3 and A2780 cells, results of Western blotting showed that the groups of PCNP overexpression had high expression level of PCNP and β‐catenin; however, PCNP knock‐down had opposite effect (Figure 6H‐I). H&E staining of serial tumour sections was shown in Figure 7A‐B, results showed that PCNP was positively correlation to tumour malignancy and metastasis. CD31 is considered to be an ideal biomarker for vascular endothelial cells. In IHC results, PCNP, Ki‐67 and CD31 expression levels were significantly reduced in the PCNP knock‐down group compared with the sh‐scb group (Figure 7C); the opposite effects were observed in the PCNP overexpression group. At the same time, we also checked the EMT‐related markers to verify the correlation between PCNP and tumour metastasis. Consistent with results above, PCNP overexpression significantly increased the levels of N‐cadherin and decreased E‐cadherin level (Figure 7C). Together, PCNP could promote OC cells proliferation and metastasis in vivo.

## 4 DISCUSSION

In our prior studies, PCNP was reported to play a vital role in growth, migration and invasion in lung cancer and neuroblastoma,^11^, ^12^ despite acting as tumour‐promoter or ‐suppressor. Ovarian cancer whose little progress has been made to improve treatment^24^ is regarded as a disease with a poor prognosis and high mortality. In this study, we expounded the role of PCNP and its potential mechanism in ovarian cancer progression.

To preliminary investigate the role of PCNP in ovarian cancer, we utilized transcriptome landscape in TCGA which covers various kinds of genomic data with clinical information to analyse the expression level in cancer tissues compared with normal tissues. The significantly higher mRNA levels first verified the positive correlation of PCNP to ovarian cancer. To further detect the protein expression of PCNP in OC, microassay was conducted and verified the consistence of protein and RNA level for PCNP. As far as we know, ovarian cancer is marked by tumour heterogeneity and in early‐stage the symptom is not facial, suggesting that it is necessary to explore novel biomarkers for diagnosis in ovarian cancer and PCNP may supply new perspective for ovarian cancer diagnosis. The up‐regulation of RNA and protein expression of PCNP in OC tissues motivated us to perform function assays to explore the effect of PCNP on ovarian cancer development and resulted in clarifying the capacity of PCNP to promote OC cells proliferation, migration, invasion and suppress cells apoptosis. Furthermore, enrichment of aberrantly expressed genes playing a vital role in cancer signalling pathways dysregulated in OC would be helpful to develop novel targeted therapeutics strategy for OC.

According to recent reports, Wnt/β‐catenin pathway has been the most well depicted in various cancers, specifically in ovarian cancer.^25^ The increased nuclear translocation of β‐catenin regarded as critical protein in Wnt pathway contributes to abnormal activation of Wnt/β‐catenin pathway.^26^ In this study, the up‐regultion of total and nuclear β‐catenin in overexpressed‐PCNP group hinted that PCNP may activate Wnt/β‐catenin pathway by fortifying β‐catenin nuclear accumulation. Using GEPIA which can employ gene expression profiling to analysis correlation of genes,^27^ herein, we first identified out the positive correlation of PCNP to Wnt/β‐catenin pathway up‐regulated genes. Together, PCNP may activate Wnt/β‐catenin pathway further regulate targeted genes expression. When there is no Wnt ligand, a destruction complex regulates β‐catenin levels. Specifically, unphosphorylated GSK3β phosphorylate β‐catenin and target the protein for ubiquitination and proteasomal degradation. The abnormally activated PI3K/Akt signal can potentially phosphorylate its downstream regulator named GSK3β,^28^, ^29^ accelerating the β‐catenin accumulation in cytoplasm and further in nuclear, resulting in cellular differentiation, division and survival.^30^, ^31^ However, Western blotting in PCNP‐overexpressed or PCNP knock‐down OC cells showed that PI3K, Akt and GSK3β protein expression and their function‐related phosphorylation levels exhibited almost no change, signifying the role of PCNP in promoting OC progression is not related to PI3K/Akt/GSK3β signal. Together, the increase of β‐catenin and the stability of its upstream signal PI3K/Akt/GSK3β manifested that the effect of PCNP on β‐catenin may be direct interaction instead of the upstream regulation. To validate the hypothesis, we conducted Co‐IP assays and clarified the interaction of PCNP to β‐catenin.

EMT is a reversible process which is tightly regulated by various transcription factors such as ZEB1.^32^ Wnt/β‐catenin signalling mediates ZEB1 expression, which induces the phenotype and cellular plasticity of EMT.^33^, ^34^ EMT is characterized by loss of the epithelial marker E‐cadherin and increased expression of mesenchymal markers, such as Vimentin.^35^ In our results, the mediated protein levels of ZEB1, E‐cadherin and Vimentin because of PCNP reflected that PCNP may regulate EMT. The morphological change of A2780 cells because of PCNP further clarified the conclusion. Current studies suggest that the up‐regulated expression of β‐catenin in the nucleus is associated with poor prognosis and metastasis.^36^ Hence, the PCNP/β‐catenin axis promotes ovarian cancer progression.

In summary, the present study here signifies that PCNP is overexpressed in OC and positively correlated with poor survival. PCNP enhances OC cell proliferation, migration and invasion by interacting to β‐catenin and prompting β‐catenin nuclear translocation, providing the first evidence for PCNP/β‐catenin network in OC (Figure 7D). Together, these data indicate that PCNP/β‐catenin signalling may be an effective and significant therapeutic target for OC treatment. However, further studies should be performed to identify the precise sites regulating the association between PCNP and β‐catenin, which will be a key to developing specific inhibitors targeting PCNP/β‐catenin signalling.

## CONFLICTS OF INTEREST

The authors confirm that there are no conflicts of interest.

## AUTHOR CONTRIBUTION

**Pengzhen Dong:** Investigation (equal); Methodology (lead); Writing‐original draft (lead). **Hao Fu:** Investigation (lead); Methodology (supporting); Software (equal). **Lin Chen:** Data curation (equal); Methodology (supporting); Software (supporting). **Shihui Zhang:** Methodology (equal). **Xin Zhang:** Methodology (supporting). **Huimin Li:** Conceptualization (lead); Data curation (lead); Methodology (supporting); Resources (lead); Writing‐review & editing (equal). **Dong‐Dong Wu:** Conceptualization (supporting); Writing‐review & editing (lead). **Xin‐Ying Ji:** Conceptualization (supporting); Funding acquisition (lead). 

## ETHICAL APPROVAL STATEMENT

This study was reviewed and approved by the Ethical Board at Henan University.

## Data Availability

The data that support the findings of this study are available from the corresponding author upon reasonable request.
